# Assessment of Milk Quality and Food Safety Challenges in the Complex Nairobi Dairy Value Chain

**DOI:** 10.3389/fvets.2022.892739

**Published:** 2022-06-08

**Authors:** Stella Kiambi, Eric M. Fèvre, Pablo Alarcon, Nduhiu Gitahi, Johnstone Masinde, Erastus Kang'ethe, Gabriel Aboge, Jonathan Rushton, Joshua Orungo Onono

**Affiliations:** ^1^Department of Public Health, Pharmacology and Toxicology, University of Nairobi, Nairobi, Kenya; ^2^International Livestock Research Institute, Nairobi, Kenya; ^3^Directorate of Veterinary Services, Nairobi, Kenya; ^4^Institute of Infection and Global Health, University of Liverpool, Liverpool, United Kingdom; ^5^Institute of Infection, Veterinary and Ecological Sciences, University of Liverpool, Liverpool, United Kingdom

**Keywords:** dairy-value-chain, Nairobi-Kenya, urban, total-bacteria, total-coliform, food-safety, challenges

## Abstract

Food networks present varying food safety concerns because of the complexity of interactions, production, and handling practices. We investigated total bacteria counts (TBCs) and total coliform counts (TCCs) in various nodes of a Nairobi dairy value chain and identified practices that influence food safety. A value chain analysis framework facilitated qualitative data collection through 23 key informant interviews and 20 focus group discussions. Content thematic analysis identified food safety challenges. Cow milk products (*N* = 290) were collected from farms (*N* = 63), collection centers (*N* = 5), shops/kiosks (*N* = 37), milk bars (*N* = 17), roadside vendors (*N* = 14), restaurants (*N* = 3), milk vending machines (*N* = 2), mobile traders (*N* = 2) and a supermarket (*N* = 1). Mean values of colony-forming units for TBC and TCC were referenced to East African Standards (EAS). Logistic regression analysis assessed differences in milk acceptability based on EAS. The raw milk from farms and collection centers was relatively within acceptable EAS limits in terms of TBC (3.5 × 10^5^ and 1.4 × 10^6^ respectively) but TCC in the milk from farms was 3 times higher than EAS limits (1.5 × 10^5^). Compared to farms, the odds ratio of milk acceptability based on TBC was lower on milk bars (0.02), restaurants (0.02), roadside vendors (0.03), shops/kiosks (0.07), and supermarkets (0.17). For TCC, the odds that milk samples from collection centers, milk bars, restaurants, roadside vendors, and shops/kiosks were acceptable was less than the odds of samples collected from farms (0.18, 0.03, 0.06, 0.02, and 0.12, respectively). Comparison of raw milk across the nodes showed that the odds of milk samples from restaurants, roadside vendors, and shops/kiosks being acceptable were less than the odds of samples collected the farm for TBC (0.03, 0.04, and 0.04, respectively). For TCC, the odds of raw milk from collection centers, restaurants, roadside vendors, milk bars, and shops/kiosks being acceptable were lower than the odds of acceptability for the farm samples (0.18, 0.12, 0.02, 0.04, and 0.05, respectively). Practices with possible influence on milk bacterial quality included muddy cowsheds, unconventional animal feed sources, re-use of spoilt raw milk, milk adulteration, acceptance of low-quality milk for processing, and lack of cold chain. Therefore, milk contamination occurs at various points, and the designing of interventions should focus on every node.

## Introduction

The global dairy industry, comprised of ~265 million cows, has continued to grow over the past decade, with milk production increasing from 590 million tons in 2009 to 683 million tons in 2018 (1). Of these, Kenya produced ~3.8 million tons, which represented about 10.8 and 0.6% of the African and global shares, respectively. While comparatively small in terms of global production, the dairy sector is significant to Kenyan livelihoods, both nutritionally and economically (2). The sector is one of the largest agricultural segments of the country, contributing about 4% of the national gross domestic product (GDP) and 14% of the agricultural GDP (3). Exponential growth of the dairy sector is expected with the predicted rise in demand for milk and other milk products influenced by growth in population, rapid urbanization, and desire for intake of livestock source foods (4). For example, Herrero et al. (4) predicted a triple increase in milk demand in Sub-Saharan Africa by 2050. Similarly, the Food and Agriculture Organization (FAO) of the United Nations forecasts a 175% rise in milk demand for Kenyans between 2010 and 2050 (5).

Increased demand for milk consumption coupled with its predicted low supply will put pressure to existing value chains, and this will trigger the evolution of more milk supply chains, which will complicate already complex food systems. Food systems present some of the most complicated networks, especially in urban areas where production and distribution are through simple to complex value chains [(6–8); Hueston and MacLeod, 2012]. Such system complexities are excellent avenues for the introduction and transmission of pathogens, including food hazards among other food safety risks (9, 10).

According to the national livestock production report of 2012, Nairobi, one of the fastest-growing urban cities in Africa (11) produced ~39 million L of milk (12). This was less than the required 388 million L based on the estimated 125 L per capita milk consumption in urban areas (13) for the 3.1 million city residents then (14). Nairobi, the largest city in Kenya and representing ~9.2% of the national population, has grown by 45% from 3.1 million people in 2009 (15) to 4.4 million in 2019 (15). This rapid population growth is expected to create a lot of pressure to produce and supply more food for city dwellers. This may trigger the evolution of more complex food chains which, in turn, may present challenges in food safety standards.

Based on the annual per capita consumption and projected growth rate of the city, approximately at 4% (11), it means that by 2050, there will be ~10.3 million residents in Nairobi and that they will require about 1.8 billion L of milk. It follows that more than 97% of milk consumed in the city will be sourced from production systems that are based outside the city. Currently, the proportion of milk that comes from outside Nairobi accounts for about 75–90% (6). The characteristics of milk chains supplying Nairobi have been described as highly complex and made up of multiple small-scale value chain actors who are highly interconnected and interdependent (16). The study by Kiambi et al. (16) described seven chain profiles (segments of the milk value chain) that form the Nairobi dairy food system. These include chain profiles for urban and peri-urban farming systems, profiles of traders affiliated to Dairy Traders Association (DTA) and those not belonging to this association (non-DTA), profiles associated with medium and large dairy cooperatives, and profiles of large processing companies. These chain profiles are tightly interlinked to form the vast Nairobi's dairy system within the free milk trading market (liberalized) (17).

Achieving food safety in such complex food systems is a challenge, particularly because milk is produced primarily by small-scale farmers, and marketing channels are dominated by informal systems (18–20). The rising milk demand (4, 5) coupled with unmatched production (4), complex interactions between value chain actors (16, 21), and compromised governance of the Nairobi dairy system (21) will put massive pressure on existing milk value chains, with a possible evolution of new ones to satisfy the rising demand. Complexities associated with urban food chains could provide excellent platforms to expand the range of food-borne pathogens as well as to amplify health and economic impacts of a single contamination incident (7). On the other hand, the degree of mixing and contact between human and livestock in urban environments has been shown to create ecological niches with opportunities for pathogen transmission, and some studies have linked urbanization to risk of emerging infectious diseases (22, 23).

There are many factors that may contribute to unsafe milk (24), and challenges in food safety in Africa are precipitated by poor food safety systems, lack of systematic surveillance, underdeveloped human resource, and insufficient capacity to determine the magnitude of the problem (25). Considering the uneven distribution of hazards/risks (26), competing priorities and inadequate resources in these countries, designing and implementation of interventions to promote food safety require a targeted risk-based approach that focuses on value chain analysis (27). This involves a thorough understanding of the “what” (e.g., contamination practices, quality deficiencies, and poor accessibility), “when” (risk seasonality), “where” (in which chains, chain nodes, areas it occurs), “who” (who creates it and who is exposed), “how” (practices and behaviors), and “how much/many” (e.g., how much contamination or how many people are exposed) (26). Several studies have mapped various value chains within food systems in Nairobi, namely, beef, sheep, and goat value chains (28), camel milk value chain (29), livestock keeping in the city (28), Nairobi dairy value chain (16), poultry value chain (8), pork value chain (30), and governance issues (21). These studies provide the critical frameworks needed for full analysis of food systems and to guide the development of necessary interventions along the value chains. In addition, such detailed scrutiny of the systems helps to understand the dynamics therein including assessment of any structural vulnerabilities (26, 28, 31).

Bacteriological characteristics of milk help to determine the quality of milk flowing through various nodes of the value chains. Bacteriological quality depends on various factors, among them is the health status of animals (32–36) as well as practices in milk handling and storage (37–39). Some studies on raw milk have demonstrated the presence of a wide range of microorganisms in marketed milk associated with poor hygiene standards (33, 40–42), or possible infection of cows in farms as shown by the presence of zoonotic pathogens like *Mycobacterium* species (33), *Brucella abortus* and *E. coli* O157:H7 (43, 44). Other hazards identified in raw milk at market level include the presence of aflatoxins (45, 46), antibiotic residues (47), and antibiotic-resistant bacteria (48). On the other hand, although pasteurized milk is expected to have minimal hazards, some studies have found excessive levels of bacteria in processed milk (19, 49), which may indicate system failure in terms of ensuring high quality of milk that arrives in processing or faulty pasteurization processes or failure in post pasteurization hygiene (50). In addition, compromised regulatory standards and procedures at pre- and post-marketing may also influence the quality of processed milk as shown in studies conducted in China on an infant formula that was adulterated with melamine (51, 52).

Investigation of bacterial load, specifically analysis of TBC and TCC, has been widely conducted as an indicator to determine the hygienic quality of milk (53). TBC in milk reflects the total number of bacteria that can grow to form countable colonies on standard methods agar when incubated aerobically at 32°C for 48 h (54). Milk stored at ambient temperatures with poor hygienic standards would favor bacterial growth and multiplication leading to its deterioration (55, 56). On the other hand, coliform bacteria are present in the environment and in the feces of all warm-blooded animals and humans (57–59). Therefore, while it is possible that infected cows could shed the bacteria in milk (60–62), detection of coliform in milk may indicate possible contamination with bacteria from the cow environment, including udder, milking utensils, water, or the handler (63).

This study overlays a bacteriological analysis onto the mapping framework developed by Kiambi et al. (16) to provide an indication of the quality and safety of milk flowing along the value chain in terms of TBC and TCC. The authors appreciate that TBC and TCC are not major public health hazards that would be present in the milk supplied to Nairobi, but their presence in higher than acceptable limits would indicate possible areas of system vulnerability and, therefore, point to critical areas of intervention. The research questions investigated in this study were: (i) how do TBC and TCC compare along the various nodes of the Nairobi milk value chain and (ii) what are the milk production and handling practices that would influence food safety along the value chain?

## Materials and Methods

**Figure 1:**
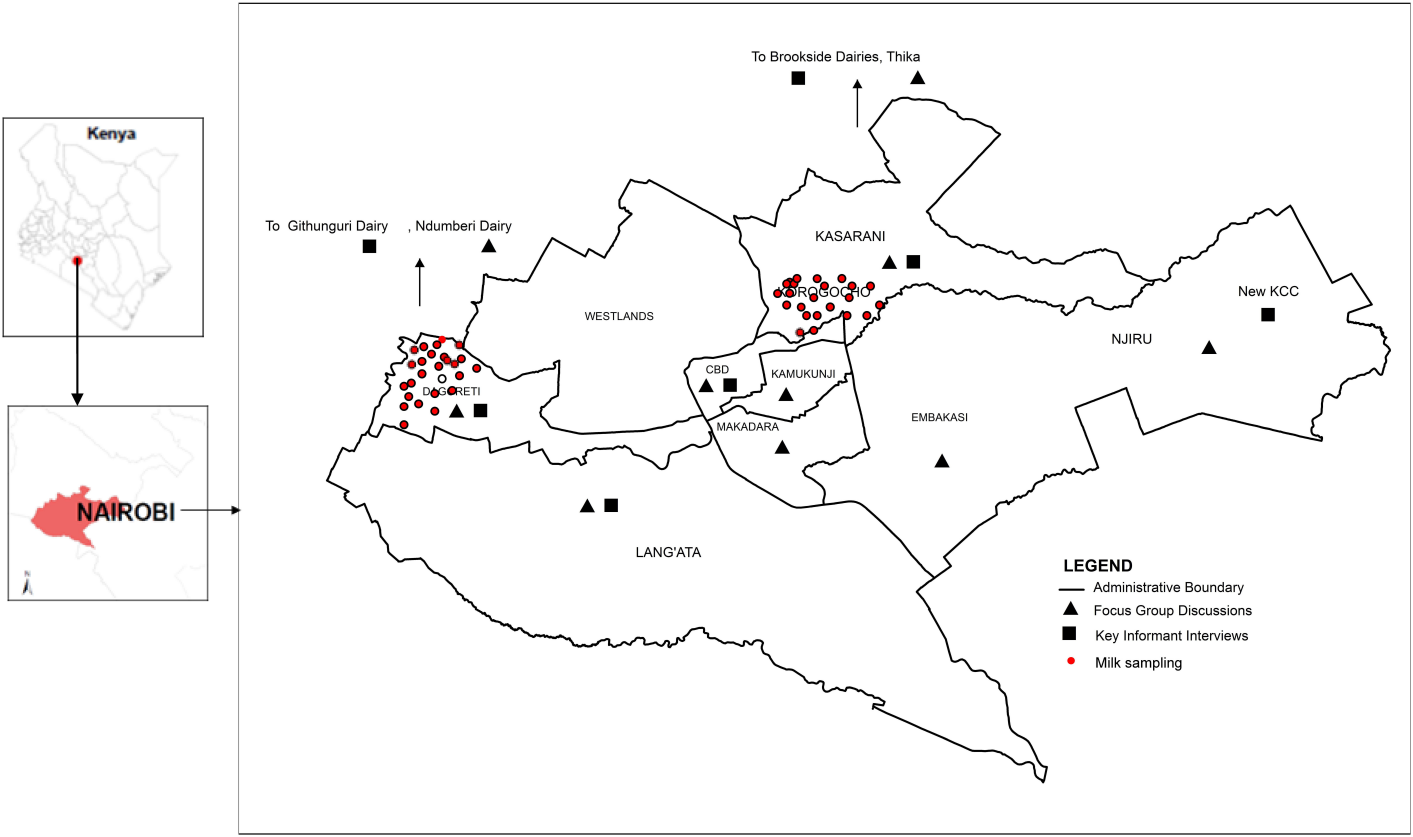
Map of Nairobi, Kenya, showing the study sites. The figure identifies areas where FGDs and KIIs were conducted and red dotted areas the sites where milk samples were obtained. Uthiru (Dagoretti) represents a peri-urban area with predominance of local milk production but also several retail chains while Korogocho (Kasarani) represents milk systems in an informal settlement area where keeping cows is not a key feature.

This study was part of the Urban Zoo project, which was based at the International Livestock Research Institute (ILRI), Kenya. The overall objective of the Urban Zoo project was to understand mechanisms leading to the introduction and transmission of pathogens to urban populations through livestock commodity value chains in 33 sub-locations of Nairobi. Most of the qualitative data used in this study were collected during the mapping of the dairy value chain (16) implemented in Nairobi County, the capital city of Kenya, between January 2014 and January 2015. The County, which is divided into nine administrative sub-counties (Figure 1), lies 696.1 km^2^ of land located at 1.2921° S, 36.8219° E and at an altitude of 1,798 m above sea level.

To comprehensively map the bacteriological (TBC and TCC) landscape of milk in Nairobi, milk sampling was conducted on nodes that were identified during the initial mapping by Kiambi et al. (16). These included farmers (within the city and from peri-urban areas), traders whether fixed (milk bars, shops/kiosks, and restaurants) or mobile (hawkers or roadside vendors), milk vending machines (MVMs), milk collection centers, and large processing companies (mainly branded processed products).

### Selection of Participants for Focus Group Discussion and Key Informants' Interviews

The selection of participants for focus group discussions (FGDs) was facilitated by government animal health assistants (AHAs) in the sub-county of focus. This, however, was in contrast to the FGD with KDB licensing officers, which were organized by the KDB head office and the Kibera FGDs, which were facilitated by a community mobilizer (a famous person involved in most health-related mobilization activities in the community). The guidance on selection of participants was given to the AHAs/community mobilizer such that for each group there was adequate gender representation, wider geographical coverage of the people (so that all participants did not come from one village), and participant's vastness in understanding of the dairy systems in the area. Each group of participants was selected based on their specific type of business/enterprise as described by the stakeholder analysis. The various FGDs that were conducted included dairy cow farmers from Kibera, an urban informal settlement), farmers in Kikuyu and Dagoretti (peri-urban areas) dairy cooperatives, traders not associated with DTA, retailers, LPOs, PHOs, KDB officers in charge of licensing and the city council of Nairobi. Key informant interviews were conducted with managers of a feed manufacturing company, officials of DTA, various managers of large processing companies (procurement of raw milk, food safety, and quality assurance), KDB, and representatives from the Directorate of Veterinary services and Department of Livestock Production.

### Data Collection in Focus Group Discussions and Key Informant Interviews

Twenty FGDs with 105 people and 23 key informant interviews (KIIs) with 35 people were conducted. From each of the FGDs, a checklist with open-ended questions was used for data collection. Qualitative data were collected on: (i) production practices including housing of the livestock, sourcing of livestock feeds, management of animal health, management of milk obtained from cows undergoing treatment, (ii) milk handling practices in bulking centers including assurance of milk safety/quality, management of milk that has been rejected for poor quality or spoilage, transportation of raw milk from farms, and processing practices that could influence food safety, (iii) practices at a retail level including sourcing of milk, transportation, value addition, management of spoilt milk, and (iv) waste management and food safety management practices. In addition, all the participants were asked to describe challenges that they or other people working in the milk system experienced that would lead to compromise in food safety. The information gathered through FGDs was triangulated during KIIs. When discrepancies were detected, additional interviews were conducted with other experts working in or conducting research on the dairy value chain.

### Epidemiological Data Collection

In addition to data from the FGDs and KIIs, epidemiological data were collected from nodes of the value chain where milk samples were collected. In each node, a pretested questionnaire was administered to collect data on: (i) type of node, (ii) amount of milk handled per day, (iii) type of milk and milk products handled, (iv) main sources of milk, (v) methods of milk preservation, and (vi) costs related to buying and selling of milk. Documentation of the interview processes was aided by video and voice recording (following consenting of the participants) and taking notes.

### Selection of Chain Nodes for Collection of Milk Samples

The selection of sampling sites for this study involved a process whereby the 33 sub-locations (where the Urban Zoo project was working) were entered in a Microsoft Excel worksheet (http://www.wikihow.com/Create-a-Random-Sample-in-Excel) to facilitate random selection of one sub-location from a peri-urban area and another from an informal settlement to represent milk chains in the two different settings. The Uthiru and Korogocho areas (Figure 1) were selected with the software. The Uthiru location is a peri-urban area with ~17,000 people (14), and rearing of dairy cows is a major activity. This area was, therefore, selected to illustrate the types of chains as well as milk quality and safety concerns in an area of relatively high milk production. On the other hand, the Korogocho location, the fourth largest informal settlement area in Kenya, had a population of ~200,000 people occupying an area of about 1.5 km^2^ (14), and livestock keeping was not a major activity. Korogocho was, therefore, selected to illustrate dairy value chains in more densely populated and lower-income areas of the city.

Listing of the different categories of dairy value chain segments (nodes) in the sub-location was aided by the area administrative officer (chief). These segments included various production units (farms), collection centers, distributors, and milk retailers. The nearest dairy farm from the chief's office was the first to be enrolled in the study upon obtaining consent. Subsequently, the next nearest farm in the same village was identified, and the procedure was repeated up to a maximum of four farms within one single area in a village. This was considered cluster one. Then, the research team moved about 200–300 m in the Uthiru area and about every 50 m in the Korogocho area (because of much higher household density) from the first cluster to another within the same village, and the process was repeated. This was done throughout the village until the teams got to the start point before proceeding to the next village where the same procedure was repeated. For retailers, up to four milk vendors identified between and within the clusters and between the villages were enrolled. These included shops/kiosks, restaurants, milk bars, roadside vendors, supermarkets, automated milk machines, milk collection centers, and mobile traders (hawkers).

### Collection of Milk Samples

A biological sampling of cow milk was conducted in farms, milk bars, shops/kiosks, supermarkets, restaurants, roadside vendors, milk vending machines, and milk collection centers. Different milk types that were sampled included: raw, pasteurized liquid milk, ultra heat treated, fermented, and yogurt. In farms, milk samples were collected only in morning hours when the temperature was cool. A farmer was requested to obtain about 50 ml of milk directly into a sterile barcoded falcon tube. However, if the farmer was unable to milk for whatever reason, they were requested to provide whatever remained from the last milking. To obtain about 50 ml of milk from the other nodes (retail and bulking centers), participants were requested to transfer the sample directly into the sterile barcoded falcon tubes. However, if the milk was in packets or sealed bottles, the entire content was purchased. All the milk samples were immediately placed in a cool box that was packed with ice packs and transported to the laboratory within 2–4 h of collection.

### Determination of Total Bacteria Count and Total Coliform Count

For the determination of TBC, samples were prepared according to the protocol described by Christen et al. (54). For each sample, 10-fold serial dilutions (10^−1^-10^−4^) were prepared in a sterile phosphate-buffered diluent (0.0425 g of potassium dihydrogen phosphate per L of distilled water), pH 7.2. Enumeration of TBC was conducted using sterile standard plate count agar (SPCA); (APHA; Oxoid^®^) that was prepared according to the manufacturer's instructions. One milliliter of an undiluted milk sample and each of the four serial dilutions were aseptically pipetted into a separate sterile pre-labeled disposable 90-mm diameter Petri dish on which freshly prepared agar was poured. The mixture (sample plus media) was gently but thoroughly mixed by whirling to ensure even distribution of the sample into the culture medium. The content was left to solidify at room temperature, and the plates were incubated at 32°C for 48 h. This was followed by an assessment of plates that had countable colony-forming units (CFUs). Plates that had between 25 and 250 CFUs per plate were selected for enumeration.

For the determination of TCC, sample preparation was carried out similarly as those for TBC, and only the first three serial dilutions were used. For enumeration of TCC, milk samples were cultured in Violet Red Bile Agar (VRBA; Oxoid^®^) guided by the manufacturer's instructions. Culture and isolation were carried out as described elsewhere (54). Incubation for coliforms was conducted for 24 h at 37°C for growth. Plates with discrete 15 to 150 CFUs were selected for counting. For both TBC and TCC, colony counting was aided with a colony counter (CLC-570).

### Data Analysis and Presentation of Results

The results are presented in a format that combines both qualitative and quantitative data. This approach was used given qualitative results often explain quantitative trends and vice versa.

#### Assessment of Food Safety Practices Through Focus Group Discussions and Key Informant Interviews

The voice and video recordings were carefully listened to, and all the information was collated in pre-formatted templates (i.e., Word documents organized to enter qualitative data in distinct sections based on predefined categories related to food safety concerns). Data entry was complemented with data collected in notebooks. The first step was to collate data in pre-formatted word documents. This allowed for systematic organization of the emerging food safety themes. The second stage of analysis entailed thorough reading of the templates and organization of the data in distinct sections based on the emerging food safety themes, which were categorized as challenges. These included a category on what practice(s) was of food safety concern, who said it (during the interview), where the practice(s) was mentioned to occur, and why the practice was said to occur. To comprehensively explore factors that may impact food quality and safety, the qualitative analysis contextualized the main practices that were mentioned in milk production, bulking centers, processing, transportation, and retailing.

#### Analysis of Data

Data cleaning, coding, and analysis were conducted in Stata 16 (64). Descriptive statistics measures, including mean, median, minimum, and maximum, were used to present the values for TBC and TCC. However, for TBC, nodes that had <3 samples were excluded from the analysis because of the impracticability of analysis of means and medians from only one or two samples. These included samples from milk vending machines (2 pasteurized), traders (2 raw), homemade yogurt from milk bars (4), homemade fermented milk (4), and yogurt from restaurants (4). These samples were not considered for further analysis. Interpretation of results for TBC and TCC was referenced to the limits specified in the East African Standards (EAS) developed in 2017 (65). These are provided in Table 1.

**Table 1:** East African Standards (2017) referenced in interpretation of Total Bacterial and Total Coliform Counts.

**Standard**	**Milk type**	**Microbiological quality limits**	**Source**
**Interpretation for total bacteria count**			
EAS 67	Raw cow milk		https://archive.org/details/eas.67.2006
	Grade I	<2 × 10^5^	
	Grade II	>2 × 10^5^–1 × 10^6^	
	Grade III	>1 × 10^6^–2 × 10^6^	
EAS 69	Pasteurized milk	3 × 10^4^	https://archive.org/details/eas.69.2006
EAS 33	Yogurt and fermented milk	0^*^	https://archive.org/details/eas.33.2006
EAS 27	Ultra-Heat Treated (UHT)	10	https://archive.org/details/eas.27.2006
**Standard**	**Milk type**	**Maximum total coliform count per ml**	**Source**
**Interpretation for total coliform count**			
EAS 67	Raw cow milk		https://archive.org/details/eas.67.2006
	Very good	0–1 × 10^3^	
	Good	1 × 10^3^-5 × 10^4^	
EAS 69	Pasteurized milk	10	https://archive.org/details/eas.69.2006
EAS 33	Yogurt and fermented milk	0	https://archive.org/details/eas.33.2006
EAS 27	UHT	0	https://archive.org/details/eas.27.2006

* *Total plate count includes yeast and molds which have a limit of 10 for E. coli, Salmonella spp. and Staphylococcus aureus*.

#### Statistical Analysis

A logistic regression analysis was performed to detect differences in milk TBC and TCC between various nodes and milk types. Two binary outcome variables were used as an indicator of whether a sample was acceptable or not based on levels according to the EAS standards (65) for TBC and TCC. Logistic regression models were then run to assess difference in the outcomes variables (1) per node (two models) (2) per milk type (two models), and (3) per node type but only considering raw milk samples (two models).

Model coefficients are reported at odds ratios (OR) where coefficients above 1 indicate increase in odds and coefficients below 1 indicate decrease in odds. Because of the clustered nature of the data (i.e., milk samples clustered in nodes), the variance-covariance matrix corresponding to the parameter estimates was specified using a clustered sandwich estimator, i.e., vce (cluster) command in Stata. This estimator allowed us to account for intragroup correlation for the estimation of standard errors. The model specification was performed in Stata 16.1 (64).

### Ethical Approval

Ethical approval for this study was obtained from the ILRI Institutional Research Ethics Committee (project reference: ILRIIREC2014–04/1). ILRI IREC is accredited by the National Commission for Science, Technology, and Innovation (NACOSTI) in Kenya. Ethical approval was also obtained from the Royal Veterinary College ethics committee (project reference: URN 2013 0084H).

## Results

### Characteristics of Participants and Milk Samples

One hundred and forty four people were interviewed during milk sampling. Of these, 56.9% (*N* = 82) were women. The age of participants ranged from 18 to 86 years, with a mean age of 41.69 and mode of 45 years. Most of the respondents (≈85%) reported to own an enterprise, while the rest were either employees (≈12%) or relatives (≈3%). For those who kept cows (farmers), the majority of people (≈84%) reared between 2 and 3 milking cows, followed by those keeping 6–9 cows (≈13%). Only a small proportion of farmers (≈3%) kept 10–13 milking cows. In terms of volumes of milk handled per day, the majority of people (≈59%) reported to handle between 0.5 and 20 L of milk, followed by 21–100 L (≈34%), while only a small proportion (≈7%) handled more than 100 L per day.

Two hundred and ninety (290) cow milk samples were collected from various nodes represented by the respondents. These included farms (*N* = 63), milk collection centers (*N* = 5), kiosks (*N* = 37), milk bars (*N* = 17), roadside vendors (*N* = 14), restaurants (*N* = 3), mobile traders (*N* = 2), milk vending machines (*N* = 2), and a supermarket (*N* = 1). The different types of samples collected included raw milk (*N* = 203), homemade fermented milk (*N* = 12), pasteurized milk (*N* = 35), ultra heat-treated milk (*N* = 13), processed yogurt (*N* = 13), and processed fermented milk (*N* = 11).

About 44% of the milk from which these samples were obtained was described to have come from within Nairobi County. The rest (≈50%) was described to be sourced from Kiambu County (peri-urban area neighboring Nairobi), about 3.4% from further rural areas, and a small proportion of milk (≈2.6%) was of unknown origin. Delivery of milk from various sources was reported to be mainly (≈88%) direct (own cows or own transport). The rest reported to have milk delivered by traders (≈9%), dairy cooperatives (≈3%), and a small proportion (≈0.5%) by processors (processed products). Information regarding recent use of antibiotics in cows from which samples were obtained was not known by 50% of the respondents, while the rest of the participants said that antibiotics had not been used in cows for about 2 weeks prior to the sampling.

### Results on Total Bacterial Counts

Table 2 displays the mean, median, minimum, and maximum parameters for the TBC of different types of milk from different nodes of the dairy value chain. The milk from production nodes (farms and collections centers) was on average within the acceptable EAS limits. The milk from farms had a mean of 3.5 × 10^5^ cfu/ml, at grade II of EAS (>2 × 10^5^–1 × 10^6^), while that from collection centers was within the limits, at grade III of EAS (1 × 10^6^–2 × 10^6^) at 1.4 × 10^6.^ This shows that the mean values for the milk from farms were better in terms of TBC than that from collection centers. Furthermore, the bacterial quality of milk deteriorated at the retail level (restaurants, milk bars, roadside vendors, and shops/kiosks). The liquid milk from all nodes except for pasteurized and UHT had higher mean values than the raw milk from farms which had a mean of 3.5 × 10^5^ cfu/ml. For example, cfu/ml mean value for the raw milk from collection centers was four times higher than the EAS limits recommended for raw milk from farms, 11.4 times for restaurants, 12.3 times for milk bars, 22.6 times for roadside vendors, and 9.4 times for milk collected from shops/kiosks. Mean values for processed (pasteurized and UHT) products were within the EAS limits. When comparing the mean values of milk samples that had values with unacceptable EAS standards, the milk from roadside vendors was worst in terms of TBC, followed by milk bars, restaurants, and shops/kiosks in that order.

**Table 2:** Total bacteria count (TBC) in milk sampled from various nodes of the Nairobi's dairy value chain.

**Variable**	**Node type**											
	**Farms**	**Collection centers**	**Restaurants**	**Milk bars**		**Roadside vendors**	**Shops/Kiosks**		**Shops/supermarket**			
**Sample type**	**Raw** **(*N* = 107)**	**Raw** **(*N* = 12)**	**Raw** **(*N* = 6)**	**Raw** **(*N* = 6)**	**Homemade fermented milk** **(*N* = 8)**	**Raw** **(*N* = 14)**	**Raw** **(*N* = 27)**	**Homemade fermented milk** **(*N* = 3)**	**UHT** **(*N* = 13)**	**Pasteurized** **(*N* = 33)**	**Fermented processed milk** **(*N* = 11)**	**Processed yogurt** **(*N* = 12)**
Mean	3.5 × 10^5^	1.4 × 10^6^	4.0 × 10^6^	4.3 × 10^6^	5.2 × 10^6^	7.9 × 10^6^	3.3 × 10^6^	3.9 × 10^6^	3.5 × 10^3^	3.1 × 10^4^	5.0 × 10^3^	4.5 × 10^4^
Median	3.3 × 10^4^	5.0 × 10^5^	3.4 × 10^6^	2.5 × 10^6^	3.5 × 10^6^	3.5 × 10^6^	1.0 × 10^6^	6.9 × 10^5^	0	0	1.2 × 10^3^	3.3 × 10^1^
Minimum	0	1.9 × 10^3^	1.6 × 10^5^	0	1.2 × 10^4^	4.6 × 10^3^	1.7 × 10^2^	1.2 × 10^4^	0	0	0	0
Maximum	6.2 × 10^6^	9.2 × 10^6^	8.8 × 10^6^	1.1 × 10^7^	2.2 × 10^7^	3.1 × 10^7^	2.1 × 10^7^	1.1 × 10^7^	4.6 × 10^4^	6.3 × 10^4^	3.8 × 10^5^	8.2 × 10^5^

*The table shows mean, median, minimum and maximum values for colony forming units per milliliter for TBC in different milk types. In red font are the TBC values that exceeded East African Standards 2017*.

### Results on Total Coliform Count

Table 3 displays the mean, median, minimum, and maximum parameters for the TCC of different types of milk from various nodes of the value chain. The mean values for all milk samples exceeded the acceptable EAS limits for TCC except for the raw milk from collection centers and pasteurized milk products. When liquid milk (raw, pasteurized, and UHT) samples were compared with what was considered “good” TCC by EAS (1 × 10^3^–5 × 10^4^), the TCC in milk from farms was three times higher, indicating unacceptable contamination of milk, which should not be sold to consumers. Quality of milk from roadside vendors was 13 times poorer than the EAS limits, 8.2 times poorer for milk from milk bars, 3.2 times poorer for milk from shops/kiosks, and 1.6 times poorer for milk from restaurants. Hence, the worst raw milk in terms of TCC when compared to EAS was from roadside vendors, milk bars, shops/kiosks, farms, and restaurants, in that order.

**Table 3:** Total coliform counts (TCC) in milk sampled from various nodes of the Nairobi's dairy value chain.

**Variable**	**Node type**											
	**Farms**	**Collection centers**	**Restaurants**	**Milk bars**		**Roadside vendors**	**Shops/Kiosks**		**Shops/supermarket**			
**Sample type**	**Raw (*N* = 107)**	**Raw (*N* = 12)**	**Raw (*N* = 6)**	**Raw (*N* = 6)**	**Homemade fermented milk (*N* = 8)**	**Raw (*N* = 14)**	**Raw (*N* = 27)**	**Homemade fermented milk (*****N** **=*** **3)**	**UHT (*N* = 13)**	**Pasteurized (*N* = 33)**	**Fermented processed milk (*N* = 11)**	**Processed yogurt (*N* = 12)**
Mean	1.5 × 10^5^	2.6 × 10^4^	7.8 × 10^4^	4.1 × 10^5^	2.0 × 10^5^	6.5 × 10^5^	1.6 × 10^5^	2.8 × 10^5^	0	2.1 × 10^1^	0.3 × 10^1^	9.6 × 10^2^
Median	0.2 × 10^1^	4.0 × 10^3^	4.7 × 10^4^	3.5 × 10^5^	5.0 × 10^4^	2.6 × 10^5^	6.1 × 10^4^	2.6 × 10^3^	0	0	0	0
Minimum	0	8.7 × 10^1^	4.1 × 10^3^	0	0.3 × 10^1^	1.1 × 10^3^	0.1 × 10^1^	0	0	0	0	0
Maximum	6.5 × 10^5^	1. × 10^5^	2.0 × 10^4^	9.8 × 10^5^	2.0 × 10^6^	3.0 × 10^6^	1.6 × 10^6^	8.4 × 10^5^	0	4.1 × 10^2^	2.7 × 10^1^	5.6 × 10^4^

*The table shows mean, median, minimum, and maximum values for colony forming units per milliliter for TCC in different milk types. In red font are the TCC values that exceeded East African Standards 2017*.

### Logistic Regression Analysis

For analysis based on the type of nodes, the reference node type was farm so coefficients represent differences between a farm and a node type. For TBC, the results show that milk samples from milk bars and restaurants were less acceptable than samples collected from farms (odds 0.02). Similarly, the odds of samples from roadside vendors, shops/kiosks, and supermarkets (0.03, 0.07, and 0.11) being acceptable were less than the odds of samples collected from farms (Table 4). For TCC, the model results indicated that the odds that the milk samples from milk collection centers were acceptable and less than the odds of samples collected from farms (odds 0.18), while the odds of milk from milk bars, restaurants, roadside vendors, and shops/kiosks were less than those of samples collected from farms (0.03, 0.06, and 0.12, respectively). There was not a major difference between the odds of milk from supermarkets and those of the milk from farms being TCC acceptable.

**Table 4:** Logistic regression analysis for total bacterial and total coliform counts by the node type where milk sample was obtained.

**Node type**	**TBC levels acceptable OR (95% CI)**	**TCC levels acceptable OR (95% CI)**
Collection centers	0.30	0.18
	(0.06–1.47)	(0.05–0.65)
Milk bars	0.02	0.03
	(0.01–0.10)	(0.01–0.10)
Restaurants	0.02	0.06
	(0.00–0.08)	(0.02–0.19)
Roadside vendors	0.03	0.02
	(0.01–0.16)	(0.00–0.08)
Shops/ kiosks	0.07	0.12
	(0.02–0.23)	(0.04–0.36)
Supermarkets	0.11	0.53
	(0.03–0.35)	(0.21–1.39)
Constant	16.83	16.83
	(5.32–53.25)	(6.48–43.73)
		
Observations	287	287

*All type of milk samples considered in this analysis. (Farm use as baseline category for the model)*.

An analysis comparing the TBC in raw milk from various nodes of the value chain showed that the odds that milk samples from restaurants and roadside vendors were acceptable were less than the odds that samples collected from farms were acceptable (0.03). Similarly, the odds that samples from milk bars and shops/kiosks were acceptable were lower than the odds of milk samples from farms (0.04 and 0.06, respectively). For TCC, the results showed that the odds that milk was acceptable from various nodes were all lower than the odds of acceptability for farm samples. That is, 0.12 for samples from collection centers, 0.12 for restaurants, 0.02 for roadside vendors, 0.06 for milk bars, and 0.05 for samples from shops/kiosks (Table 5).

**Table 5:** Logistic regression analysis for total bacterial and total coliform counts comparing raw milk samples from various nodes of the value chain.

**Node type**	**TBC levels acceptable OR (95% CI)**	**TCC levels acceptable OR (95% CI)**
		
Collection centers	0.30	0.18
	(0.06–1.47)	(0.05–0.65)
Restaurants	0.03	0.12
	(0.01–0.11)	(0.01–0.94)
Roadside vendors	0.03	0.02
	(0.01–0.16)	(0.00–0.08)
Milk bars	0.04	0.04
	(0.01–0.19)	(0.01–0.18)
Shops/ kiosks	0.06	0.05
	(0.01–0.22)	(0.02–0.16)
Constant	16.83	16.83
	(5.32–53.26)	(6.48–43.74)
		
Observations	201	201

*(Note: no raw milk samples obtained from supermarket). Farm used as baseline category for the model*.

For the analysis based on type of milk, the reference was raw milk (Table 6). There were few differences in the odds of TBC and TCC acceptability across milk types. For TBC, the odds that homemade milk (yogurt and fermented) and processed (yogurt and fermented) were acceptable was 0.03 and 0.1 lower, respectively, than those of raw milk. Results of the analysis of TCC showed that only the homemade (yogurt and fermented) had lower odds (0.06) of being acceptable than raw milk.

**Table 6:** Logistic regression analysis for total bacterial and total coliform counts by the milk type.

**Milk type**	**TBC levels acceptable OR (95% CI)**	**TCC levels acceptable OR (95% CI)**
Home-made (yogurt and fermented)	0.03	0.06
	(0.00–0.21)	(0.01–0.28)
Processed (yogurt and fermented)	0.10	1.15
	(0.02–0.50)	(0.58–2.28)
UHT	0.82	
	(0.41–1.65)	
Pasteurized	1.51	1.92
	(0.62–3.69)	(0.77–4.75)
Constant	2.74	2.61
	(1.86–4.03)	(1.81–3.76)
		
Observations	290	277

*Raw milk used as baseline category for the model*.

### Practices That May Influence Food Safety Along the Nairobi Dairy Value Chain

There were several practices that were mentioned during key informant interviews and FGDs that could possibly influence food safety (Table 7). In production, such factors were related to keeping cows in very muddy cowsheds, obtaining animal feed from dumpsites and market leftovers, obtaining feeds by the roadside, treatment of cows by unqualified personnel coupled with compromise in withdrawal periods following treatment, resale of milk that has been rejected from dairy cooperatives or allowing it to ferment further (and taken as fermented milk), and addition of water to increase the volume of milk.

**Table 7:** Among the practices mentioned by stakeholders as influencing food safety along the dairy value chain in Nairobi, Kenya.

**Practice(s)**	**Who said**	**Where is the practice done**	**Why the practice was said to be done**
Poor drainage, muddy cowsheds	All FGDs with farmers	Farms	- Inadequate land for expansion - Lack of capital to build good sheds with drainage
Animal feeds sourced from dumping sites, sewer lines, market leftovers, roadsides	FGD with farmers in the urban informal area	Farms	- Feed scarcity especially in dry seasons - Pasture from sewer lines is ever green and available
Mixing of sweepings from poultry houses with dairy commercial feeds	Farmers (Both FGDs in peri-urban area)	Farms	- Perceived to increase milk production
Self-treatment or use of untrained personnel for management of animal diseases	All FGDs with farmers, FGDs with LPOs	Farms	- Inadequate money to engage professionals - Unaware on where the animal health professionals were - Fear of being arrested for keeping livestock which is perceived outlaw (urban informal area)
Failure to observe withdrawal periods following use of antibiotics in milking cows	All FGDs with farmers, FGDs with LPOs	Farms	- Economic losses with milk disposal - Lack of knowledge on withdrawal
Adulteration of milk through addition of water	All FGDs with farmers, KII with DTA, FGDs with non-DTA traders and with trailers	Farms, by traders, roadside vendors, milk bars and shops/ kiosks	- To increase milk volumes especially in dry seasons when milk production is low
Adulteration of milk by adding substances like hydrogen peroxide, formalin, caustic soda, egg yolk, margarine, sugar, wheat flour	KII with DTA & FGD with non-DTA traders, FGD with trailers	By traders	- Hydrogen peroxide and formalin as preservatives caustic soda, egg yolk, margarine, sugar and wheat flour to increase milk density
Conversion of raw milk that has “accidentally” curdled to home-made “fermented milk” or “yogurt” or selling it at cheaper price	All FGDs with farmers, KII with DTA, FGDs with non-DTA traders and with trailers	By traders, farmers, milk bars, shops/kiosks, restaurants	- Believe that curdled milk is not spoilt milk. One farmer said, “*unboiled milk that curdles is very good for eating ugali*.” Ugali is a type of meal made of ground corn.
Re-sale of milk that has been rejected at the milk bulking sites	All FGDs with farmers, KII with DTA & FGD with non-DTA traders	By farmers, traders, milk bars, shops/ kiosks, restaurants	- Disposing milk is a loss (economic related factors) - There is always a ready market for such milk
Occasionally, acceptance of milk that should be rejected	FGD and KII with dairy cooperatives, KII -large processing companies	Milk collection centers, dairy cooperatives, large processors	- Milk is scarce and there is a ready milk market - If they rejected, the milk would be sold to competitors
Most milk collection centers located by roadsides and without sheds and lack of coolers	KIIs with dairy cooperative and large processing companies, FGDs with dairy cooperatives	Milk collection centers, dairy cooperatives, traders, milk bars, shops	- Low milk volumes do not warrant investment on construction of sheds - High cost of running cooling systems
Storage of milk in non-food grade plastic containers	FGD with non-DTA traders, KII – DTA traders, FGD – retailers, KII (KDB, PHOs)	By traders, milk bars, shops/ kiosks, restaurants	- Plastic containers were affordable - They were easy to transport - Have minimal spillage
Lack of training on hygiene across the value chain	All FGDs with farmers, FGDs with LPOs, KDB	By farmers, traders, milk bars, shops/ kiosks, restaurants	- There are no such trainings offered by the government on regular basis and extension services are negligible
Selling of milk through hawking from place to place or by the roadside	FGD with KII - DTA traders, KIIs with dairy cooperative and large processing companies, FGDs with dairy cooperatives, KII (KDB, PHOs)	By traders, roadside vendors	- It is cheaper to start a business informally as there are minimal capital requirements (one just needs a container and the initial milk to start the business). It is expensive to meet the formal requirements (premise, licenses)
Selling of milk in unlicensed premises	KIIs with dairy cooperative and large processing companies, FGDs with dairy cooperatives, KII –KDB	By traders, milk bars, shops/ kiosks, restaurants	- It is expensive to meet the formal requirements (premise, licenses)

*FGD, Focus group discussions; KII, Key informant interviews; DTA, Dairy Traders Association; KDB, Kenya Dairy Board; PHOs, Public Health Officers; LPOs, Livestock Production Officers*.

In bulking centers, the FGD and KII with dairy cooperatives and some large processors reported that they sometimes, especially during milk scarcity, accepted milk that should be rejected. They argued that rejection of milk in such periods of scarcity and in the midst of the liberalized dairy sector would set their competitors at an undue advantage of selling what they rejected. One of the managers in the bulking centers argued that bad milk would be neutralized (unacceptable contents would be diluted) by good milk. He said, “*since not every farmer will have bad milk or will have used antibiotics at the farm, the good milk will neutralize the bad milk, and overall all the milk will be fairly good. So we don't reject all that need to rejected except when it is grossly curdled or dirty. Our competitors who don't care about quality, especially the informal traders will be waiting for it, and they will sell it, since milk market is ever ready*.” Milk cooling and basic screening tests were said to be lacking in most collection centers, with screening relying on organoleptic tests. On disposal of milk that had been rejected in bulking centers, dairy cooperatives and large processors reported that such milk were sent back to suppliers, and they reported that some of it was returned back to the food chain.

In retail, milk adulteration by addition of water was reported to be a frequent occurrence aiming at increasing the volume of milk. This was reported to occur mainly during dry seasons when milk production was low. According to traders and retailers, the practice was mentioned to occur at the farm level, in traders (those selling milk to retailers), retailers, including roadside vendors, milk bars, restaurants, and in shops/kiosks. Another food safety challenge mentioned in retail was the lack of cooling facilities during transportation and at sale points. Raw milk that got spoilt either in transit or at sale points was said to be sold either as fresh liquid milk at a price than lower than that of raw milk, or was converted into yogurt or fermented milk. Fermented milk is basically prepared by letting the raw milk that had curdled to stay for a few more days in a container to ferment more, while the preparation of yogurt entailed the addition of flavors and color to the raw fermented curdled milk. Finally, there was a glaring gap in regulation and enforcement of food safety practices in the value chain, as several businesses (traders and retailers) were reported and observed to be operating without necessary government permits and licenses.

## Discussion

By 2050, the demand for milk consumption will triple in Sub-Sharan Africa (4), with consumption projected to rise by 175% from 2010 to 2050 in Kenya (5). The current population in Nairobi (15), coupled with high per capita milk consumption (13) that is unmatched with production has resulted in most of milk being sourced from production systems that are based outside the city (6, 16). The situation is expected to worsen with the projected population growth (11) and increased demand for milk by city dwellers (5). Food chains associated with urban food systems are complex and are, thus, likely to provide excellent platforms to expand the range of food-borne pathogens (7, 22, 23).

This study utilized the previously developed framework of Nairobi's dairy value chain (16) to investigate milk quality and safety in complex food systems to identify food safety challenges. The mapped framework demonstrated the vastness and complexity of Nairobi's dairy value chain with multiple interactions between various actors and nodes of the value chain. Based on these complex interactions, the study concluded that a holistic approach would be required to address any intervention and policy decision. This approach has been supported by other studies (8, 26, 28, 29).

This study found that TBC levels in production nodes (farms and collections centers) were generally good and within the acceptable limits of the EAS for TBC, but that TCC limits were higher (than EAS) in farms. This agrees with several other studies that have demonstrated good bacterial quality of milk at farm levels (35, 55, 66, 67). However, this could be compromised by other factors like health status of the animals (32–36) and practices related to milk handling and storage practices (37–39). Apart from TBC and TCC, which only serve as an indicator of the robustness of the system in this study, some of the practices reported (and observed by the researchers) on farms warrant further investigation to inform broader interventions. For example, the availability of animal feeds was reported to be a challenge, and sometimes farmers fed cows with feeds sourced from dumping sites, leaking sewer lines, market leftovers, and obtained by the roadside. Such unconventional feed sources may present opportunities for the introduction of heavy metals and subsequent public health concerns (68–70). In addition, wastes swept from poultry houses were reported to be mixed with dairy commercial concentrate feeds, as this was perceived to increase milk production. This practice may result in the introduction and transmission of antimicrobial resistance (AMR), as several studies have demonstrated high levels of AMR in poultry and poultry environments (71–73).

We noted that the bacterial quality of milk deteriorated at retail level. For example, the logistic regression analysis for TBC showed that the odds that milk samples from retailers were acceptable were less than the odds of samples collected from farm as follows: 0.02 (milk bars), 0.02 (restaurants), 0.07 (shops/kiosks), 0.03 (roadside vendors), and 0.11 (supermarkets). For TCC, the model results indicated that the odds of milk samples from milk collection centers were acceptable were less than the odds of samples collected from farms (0.18), and that the odds of milk from milk bars and restaurants were less than the odds of farm samples (0.03 and 0.06, respectively). Similarly, the odds of samples from roadside vendors and shops/kiosks being acceptable were less than those of samples collected from farms (0.02 and 0.12, respectively). Our results agree with findings from other studies that show such bacterial deterioration of milk as it flows from farms through the retailing system (33, 35, 42, 74). Such deterioration may be influenced by, among other factors, heavy bacterial load in source (66, 67), adulteration (75), flow of milk over longer distances without cold chain, and poor handling practices during transportation and storage. In this study, there are some reported practices that may contribute to increased bacterial count in milk at retail level. For example, milk was stored in plastic (non-food grade) containers, and cold chain was deficient as reported in various KIIs and FGDs (see Table 7). Traders and retailers in Nairobi reported to depend on local agents based at the farms to source for milk on their behalf, which was then bulked into 20-L plastic containers and transported while stack under passengers' seats in public vehicles (FGDs with non-DTA traders and retailers, KII with DTA traders). Despite the law requiring milk transportation to be done in cold chain and by licensed vehicles (transport permit issued by KDB), the traders and retailers indicated that it was expensive to travel to farms daily to source for milk as well as to own or hire a vehicle to transport their milk. Probably, that is the reason some traders mentioned to use unorthodox methods for preservation of milk like the addition of hydrogen peroxide and formalin. Malicious addition of formalin and hydrogen peroxide has been mentioned as a way of increasing the shelf life of milk in the absence of cold chain (75). Apart from ethical aspect, researchers have reported that addition of artificial preservatives, such as chemicals, could result in serious health problems, including cancer (76, 77).

If the raw milk spoilt, it was reported as “*accidentally curdled*,” and it was reported to be converted into fermented milk (allowed to ferment for several more days) or yogurt (addition of flavors and colors to the fermented milk). This probably explains the high TBC and TCC in homemade fermented milk and homemade yogurt. On the regression analysis, our results indicated that the odds that the homemade (yogurt and fermented) and processed (yogurt and fermented) types of milk were acceptable for TBC was 97 and 90% lower, respectively, than raw milk, while for TCC, the homemade (yogurt and fermented) had lower odds (94%) than raw milk. While most Kenyans normally boil milk before consumption (78, 79), it is important to note that these products (home-made fermented and yogurt) were made from raw milk that had gone bad. Consequently, these could pose significant public health threats if the raw milk was sourced from cows infected by any zoonotic disease. For example, the consumption of raw milk has been associated with brucellosis in humans (80).

The logistic regression analysis of the processed milk products showed that they were generally within the acceptable limits for TBC and TCC. This agrees with other studies that have shown the importance of pasteurization and ultra-heat treatment in significantly reducing bacterial load in milk (81–83). However, other studies have shown significant levels of bacteria in pasteurized milk (19, 49, 84–86). This happens with poor quality of raw milk (35, 55, 66, 67) when there is a breakdown in the pasteurization process(50), or factors related to post-processing handling. In this study, the assessment of maximum values for the colony-forming units for TBC and TCC showed that there were some processed milk products that were highly contaminated (refer to Tables 2, 3 in the results section). These included pasteurized milk (4.1 × 10^2^), processed fermented milk (2.7 × 10^1^), and processed yogurt (5.6 × 10^4^). Some practices elucidated during FGDs and KIIs with the dairy cooperatives and large processing companies that may contribute to high bacterial load in processed milk included lack of cold chain in collection centers and acceptance of milk that should be rejected so as not to benefit competitors who care less about quality. Unfair competition among value chain actors has been identified as one major factor that hinders achieving optimal food safety in the Nairobi dairy value chain (21, 84). Other factors that were mentioned in this study included lack of training on hygiene across the value chain, selling of milk by hawking from place to place or by the roadside, and selling of milk in unlicensed premises (hence such milk is not monitored by regulatory authorities). Therefore, addressing food safety requires concerted efforts of every actor in the value chain. Some strategies that have been deployed to improve milk safety elsewhere include improvement of infrastructures in farms and at collection, enhanced information on production, and improved frequency of milk collection to reduce build-up of bacteria in milk (87, 88). Undoubtedly, improved training programs and provision of extension services would enhance good practices in milk production and handling.

### Limitations of the Study

The first limitation that is important in interpretation of the results from this study is that milk samples were collected only in the morning when the temperature was cool and probably a few minutes or hours after milking. The bacteriology results may, therefore, not be indicative of the scenario after many hours post milking. The second limitation was that this study did not test the presence and burden of other food safety hazards that were mentioned during data collection, such as hydrogen peroxide and formalin residues. As such, this may have limited our understanding of the true burden of public health concerns. However, we have attempted to describe some practices along the value chain that may trigger further research. Finally, our sample size was only restricted to Nairobi, so we cannot generalize these statements to the entire Kenyan dairy value chain. In addition, the sample size was relatively small for many sample types; therefore, the results cannot be generalized to the entire Nairobi dairy value chain. However, the initial assessments are critical in suggesting domains for broader assessments and testing methods to better understand the dairy food system. For the qualitative data that were gathered mainly through narrations in FGDs and KIIs, we indicated which theme was mentioned by what type of stakeholder (Table 7), but information on how many stakeholders in an FGD or KII mentioned or supported a particular theme was not collected. However, the wide variety of people represented for various segments of the value chain would help to point out areas requiring further interrogation to inform interventions.

## Conclusion and Recommendations

This study provides a detailed analysis of food quality and safety challenges in Nairobi's complex dairy value chain. The descriptive analysis of the mean values for TBC and TCC shows that raw milk collected from farms and processed products were relatively good, but these parameters deteriorated at the retail level. The logistic regression analysis on all sample types showed that the odds that milk samples from retail (milk bars, restaurants, shops/kiosks, roadside vendors, and traders) were acceptable were less than the odds of samples collected from farm being acceptable for TBC and TCC. Likewise, the odds that raw milk samples from retail (milk bars, restaurants, shops/kiosks, roadside vendors, and traders) were acceptable was less than the odds of raw samples collected from farm being acceptable for TBC and TCC. With raw milk as the reference, the analysis by milk type showed few differences in the odds of TBC and TCC acceptability across milk types.

Several practices with possible influence on food safety were mentioned. In production, these were related to keeping cows in very muddy cowsheds, obtaining animal feeds from dumpsites and market leftovers, obtaining feeds by the roadside, treatment of cows by unqualified personnel coupled with compromise on withdrawal periods following treatment, resale of milk that has been rejected from dairy cooperatives or allowing it to ferment further (and taken as fermented milk), and addition of water to increase the volume of milk. In bulking centers, the practices were related to accepting milk that should be rejected, lack of cold chain and basic screening tests, and lack of procedures for the management of milk that has been rejected. In retail, there was milk adulteration by addition of water and other chemicals, lack of cooling facilities during transportation and at sale points, sale to consumers of raw milk that got spoilt, and conversion into yogurt or fermented milk.

The analytical methodology presented in this study demonstrates a practical approach for strategic policy decisions. To achieve milk quality and safety, the authors suggest the implementation of more robust training of people involved in the milk system. However, this needs to be guided by a critical analysis of prevailing challenges in every segment of the value chain. Every node of the value chain should be considered prior to designing and implementing any intervention, as further underscored in Kiambi et al. (16), FAO (26), and Alarcon et al. (28). The framework and findings obtained can help future research and policymakers to reach an informed decision on possible points of intervention in improving the quality and safety of milk flowing through complex value chains.

## Data Availability Statement

The datasets presented in this study can be found in online repositories. The names of the repository/repositories and accession number(s) can be found below: Data repository of the University of Liverpool available at https://doi.org/10.17638/datacat.liverpool.ac.uk/1639.

## Author Contributions

SK, EF, PA, JR, and EK designed the study and data collection tools. SK and JM collected data. SK, JO, PA, and EF drafted the manuscript. SK, GA, and NG developed the culture and isolation standard procedures, facilitated culture and isolation and interpreted TBD and TCC results. All authors read, commented on, and approved the final manuscript for publication.

## Funding

This study was supported by the United Kingdom Medical Research Council, Biotechnology and Biological Science Research Council (United Kingdom), the Economic and Social Research Council (United Kingdom), the Natural Environment Research Council (United Kingdom), through the Environmental & Social Ecology of Human Infectious Diseases Initiative (ESEI), grant reference: G1100783/1. This study also received support from the CGIAR Research Program on Agriculture for Nutrition and Health (A4NH) led by the International Food Policy Research Institute (IFPRI).

## Conflict of Interest

The authors declare that the research was conducted in the absence of any commercial or financial relationships that could be construed as a potential conflict of interest.

## Publisher's Note

All claims expressed in this article are solely those of the authors and do not necessarily represent those of their affiliated organizations, or those of the publisher, the editors and the reviewers. Any product that may be evaluated in this article, or claim that may be made by its manufacturer, is not guaranteed or endorsed by the publisher.

## Abbreviations

CFU: Colony Forming Unit
DTA: Dairy Traders Association
EAS: East African Standards
FGD: Focus Group Discussions
KII: Key Informant Interviews
TBC: Total Bacteria Count
TCC: Total Coliform Count.
